# Relations between character strengths, school satisfaction, enjoyment of learning, academic self-efficacy, and school achievement: An examination of various aspects of positive schooling

**DOI:** 10.3389/fpsyg.2022.826960

**Published:** 2022-10-13

**Authors:** Marco Weber, Claudia Harzer

**Affiliations:** Department of Psychology, Faculty of Human Sciences, Medical School Hamburg, Hamburg, Germany

**Keywords:** school satisfaction, enjoyment of learning, academic self-efficacy, school achievement, positive schooling, engine model, character strengths

## Abstract

This study is embedded in the theoretical framework of the engine model of positive schooling. Accordingly, relations were investigated between students’ endogenous input variables (i.e., character strengths), process variables (i.e., school satisfaction, enjoyment of learning, and academic self-efficacy), and school achievement as an outcome variable. A sample of 300 students (between 10 and 17 years of age) completed web-based self-report measures for all key variables. Specific character strengths (e.g., love of learning, zest, hope, perseverance, and perspective) were substantially positively related to school satisfaction, enjoyment of learning, academic self-efficacy, and/or school achievement. Exploratory mediation analyses supported the basic assumption that processes (i.e., school satisfaction, enjoyment of learning, and academic self-efficacy) mediate the relations between character strengths as input variables and school achievement as an outcome variable. The findings underline the benefit of studying inputs, processes, and outcomes simultaneously to better understand the interplay among relevant variables in the context of positive schooling.

## Introduction

The present research was designed to investigate the interplay among different types of variables expected to be relevant for positive schooling. Positive schooling considers both traditional academic aspects like mastering school tasks and non-academic aspects like students’ character strengths, satisfaction, and enjoyment at school (e.g., Harzer et al., 2021).

**FIGURE 1:**
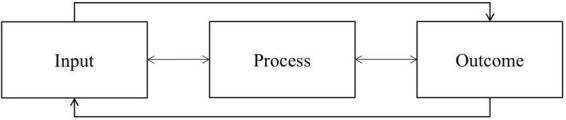
Components of the engine model of positive schooling and possible interrelations (Harzer et al., 2021).

Harzer et al. (2021) present one way to study the aforementioned interplay in a structured way. They describe an engine model of positive schooling, based on Jayawickreme et al.’s (2012) original framework of an “engine model of wellbeing.” Such an engine model distinguishes among input variables, process variables, and outcome variables (Jayawickreme et al., 2012). Inputs can be both exogenous and endogenous in nature. For example, exogenous inputs include individuals’ income or political safety, while endogenous inputs include individuals’ personality traits (Jayawickreme et al., 2012). Processes include internal states or mechanisms like feelings (moods, emotions, affect) and cognitive evaluations (e.g., global life satisfaction). Such processes affect individuals’ choices, and in turn lead individuals to exhibit specific intrinsically motivated and valuable behaviors (i.e., outcomes), like being engaged and acting meaningfully (Jayawickreme et al., 2012). These components may be reciprocally related to each other (see Figure 1; Harzer et al., 2021).

Harzer et al. (2021) adapted Jayawickreme et al.’s (2012) wellbeing specific approach in order to describe relevant aspects of positive schooling broadly construed (Harzer et al., 2021). For example, exogenous input variables in the schooling-related engine model include student-teacher relationships, parental involvement in schooling, peer relationships, perception of safety, and teachers’ instructional behaviors (Harzer et al., 2021). Personality variables like self-esteem, hope, locus of control, and character strengths are seen as endogenous input variables (Harzer et al., 2021). Process variables include cognitive evaluations of circumstances and competences like school satisfaction and academic self-efficacy beliefs, respectively, as well as feelings at school (Harzer et al., 2021). Students’ behaviors are described as outcome variables and can include academic performance and school achievement, engagement at school, or participation in structured extracurricular activities (Harzer et al., 2021). The present study sought to examine the relations between (a) character strengths as endogenous input variables; (b) school satisfaction, enjoyment of learning, and academic self-efficacy as process variables; and (c) school achievement as an outcome variable and widely accepted marker of school success. By examining these relations, the present paper provides first exploratory testing of some basic assumptions presented in the engine model of positive schooling (i.e., input variables relate to process variables and outcome variables; input variables related to outcome variables mediated by process variables; see Harzer et al., 2021).

### Definition of key variables

*Character strengths* are defined as positive, morally valued, cross-culturally desirable personality traits. Such characteristics are valued in their own right, and exhibiting them by individuals does not diminish others around them (Peterson and Seligman, 2004). As stable traits character strengths manifest in individuals’ behaviors (e.g., finishing what one starts), thoughts (e.g., appreciating close relationships), and feelings (e.g., being grateful for help from others). They contribute to a satisfied, fulfilling, and successful life (e.g., Peterson, 2006), which is in line with the idea of being endogenous inputs in an “engine model” (e.g., Jayawickreme et al., 2012; Harzer et al., 2021). In their Values in Action (VIA) classification of strengths, Peterson and Seligman (2004) present a multidimensional approach identifying 24 character strengths, all of which are useful for describing various aspects of a good character. The VIA classification contains six different sets of character strengths, which are grouped together theoretically based on their content (e.g., Peterson and Seligman, 2004) (see Table 1).

**TABLE 1:** 24 character strengths included in the Values in Action classification (Peterson and Seligman, 2004).

***(I) Cognitive character strengths that entail the acquisition and use of knowledge***
(1) Creativity: Thinking of novel and productive ways to do things (2) Curiosity: Taking an interest in all of ongoing experience (3) Judgment: Thinking things through and examining them from all sides (4) Love of learning: Mastering new skills, topics, and bodies of knowledge (5) Perspective: Being able to provide wise counsel to others
***(II) Emotional character strengths that involve the exercise of will to accomplish goals in the face of opposition, external or internal***
(6) Bravery: Not shrinking from threats, challenges, difficulty, or pain (7) Perseverance: Finishing what one starts (8) Honesty: Speaking the truth and presenting oneself in a genuine way (9) Zest: Approaching life with excitement and energy
***(III) Interpersonal character strengths that involve “tending and befriending” others***
(10) Capacity to love and be loved (short: love): Valuing close relations with others (11) Kindness: Doing favors and good deeds for others (12) Social intelligence: Being aware of the motives and feelings of oneself and others
***(IV) Civic character strengths that underlie healthy community life***
(13) Teamwork: Working well as member of a group or team (14) Fairness: Treating all people the same in accordance with notions of fairness and justice (15) Leadership: Organizing group activities and seeing that they happen
***(V) Character strengths that protect against excess***
(16) Forgiveness: Forgiving those who have done wrong (17) Modesty: Letting one’s accomplishments speak for themselves (18) Prudence: Being careful about one’s choices; not saying or doing things that might later be regretted (19) Self-regulation: Regulating what one feels and does
***(VI) Character strengths that forge connections to the larger universe and provide meaning***
(20) Appreciation of beauty and excellence [short: appreciation]: Noticing and appreciating beauty, excellence, and/or skilled performance in all domains of life (21) Gratitude: Being aware of and thankful for the good things that happen (22) Hope: Expecting the best and working to achieve it (23) Humor: Liking to laugh and joke; bringing smiles to other people (24) Spirituality: Having coherent beliefs about the higher purpose and meaning of life

*School satisfaction* is defined as students’ cognitive evaluations of the quality of their school experiences. Such evaluations are based on students’ comparisons of their circumstances in this specific, major life domain with the standards they have set for themselves individually (e.g., Huebner, 1994; Diener et al., 1999). School satisfaction (like life satisfaction in general) is no longer seen as an epiphenomenon, as it affects a variety of important outcomes at school (e.g., Huebner et al., 2014). This is reflected in Harzer et al.’s (2021) engine model of positive schooling, where school satisfaction is defined as a process variable and not as an outcome variable. For example, higher levels of school satisfaction were in line with lower levels of disengagement from schooling as well as lower levels of internalizing and externalizing behaviors (e.g., Harzer et al., 2021). Results on the relations between school satisfaction and school achievement are quite mixed. Some studies suggest a small but significant positive correlation between school satisfaction and school achievement, whereas others do not (see Huebner et al., 2014 for a review). Hence, more research is needed to further our understanding of the relation between school satisfaction and school achievement.

*Enjoyment of learning* is defined as a positive emotion associated with achievement-related tasks in the context of schooling (e.g., Pekrun, 2014). The specific emotion of enjoyment of learning is seen as an enabling factor for aspects such as students’ learning motivation, appropriate learning strategies, self-regulation, and ultimately academic achievement (e.g., Pekrun, 1993, 2014); all of these aspects are highly relevant for positive schooling experiences. Positive emotions (e.g., joy and happiness) have been found to be not only an outcome of actual life circumstances but are also seen as important mechanisms leading to life success (e.g., Lyubomirsky et al., 2005). For example, in a longitudinal study, math-related positive emotions (e.g., enjoyment and pride) were reciprocally related to school achievement (Pekrun et al., 2017). This is in perfect alignment with Harzer et al.‘s (2021) engine model of positive schooling, where processes and outcomes are seen as reciprocally linked with one another.

*Academic self-efficacy* is based on Bandura’s (1997) concept of self-efficacy, which is defined as “people’s beliefs in their capabilities to produce desired effects by their own actions” (Bandura, 1997, p. vii). Academic self-efficacy beliefs represent expectations of competence in dealing with diverse requirements and challenges at school specifically. Studies have shown that academic self-efficacy, which is defined as a process variable in the engine model of positive schooling (Harzer et al., 2021), affects students’ motivation at school, effective learning, and ultimately academic achievement (e.g., Schunk and Dibenedetto, 2014; Honicke and Broadbent, 2016).

### Relevant findings from prior studies

Although research on the relations among character strengths and school satisfaction, enjoyment of learning, academic self-efficacy, and school achievement is relatively sparse at the moment, some relevant empirical findings are available. On a more global level, prior research has shown that character strengths are positively related to global life satisfaction (e.g., Weber et al., 2013; Ruch et al., 2014; Weber, 2021), positive feelings in general (Van Eeden et al., 2008; Weber et al., 2013; Weber, 2021), and general self-efficacy beliefs (Ruch et al., 2014). On a more specific level, different character strengths have been found to be associated with different schooling-related variables (e.g., Park and Peterson, 2006; Weber and Ruch, 2012; Shoshani and Slone, 2013; Wagner and Ruch, 2015; Weber et al., 2016; Weber, 2018). For example, the character strengths love of learning, zest, gratitude, perseverance, and curiosity were positively related to school satisfaction (Weber and Ruch, 2012). Furthermore, the character strengths zest, love of learning, perseverance, and social intelligence were positively related to a set of school-related positive feelings like feeling active, happy, interested, and proud at school (Weber et al., 2016). Character strengths like hope, love of learning, perseverance, and prudence were positively related to academic self-efficacy (Weber and Ruch, 2012). Specific subsets of character strengths, such as character strengths of the mind or intellectual character strengths (e.g., perseverance, self-regulation, and love of learning) were positively related to school achievement (Park and Peterson, 2006; Weber and Ruch, 2012; Shoshani and Slone, 2013; Wagner and Ruch, 2015). Furthermore, first research is available on the interplay among inputs, processes, and outcomes, showing that specific character strengths (e.g., zest and perseverance) are positively related to overall school achievement mediated by positive feelings at school (Weber et al., 2016). All in all, there is first evidence that the key variables of the present study play a meaningful role in the schooling context; however, more research is needed to understand their associations in a more nuanced manner.

### The present study

One major goal of this study was to gain more knowledge on the generalizability of the aforementioned results. Therefore, character strengths’ relations with school satisfaction, academic self-efficacy, and school achievement were of interest. Moreover, in the present study, school satisfaction was measured using a psychometrically sound multi-item measurement (i.e., Multidimensional Students’ Life Satisfaction Scale; Huebner, 1994), in contrast to the one-item assessment of school satisfaction reported by Weber and Ruch (2012).

In addition, new knowledge will be gained by studying the relations between character strengths and enjoyment of learning, which is helpful for achieving a more comprehensive understanding of positive schooling’s nomological network. Furthermore, and also relevant for generalizability, the present study investigates a German sample. This adds new cross-national insights to those stemming from an American sample (Park and Peterson, 2006), an Israeli sample (Shoshani and Slone, 2013), and Swiss samples (Weber and Ruch, 2012; Wagner and Ruch, 2015; Weber et al., 2016).

As another major goal, the present study focused on the mediating role of three different process variables (i.e., school satisfaction, enjoyment of learning, and academic self-efficacy) with respect to the relations between character strengths (inputs) and school achievement (outcome). In doing so, the present study sought to yield further empirical evidence for the engine model of positive schooling (Harzer et al., 2021). This study was guided by the following four research questions:

(1) What are the relations between character strengths (Peterson and Seligman, 2004) as endogenous input variables and school satisfaction, enjoyment of learning, academic self-efficacy as process variables? As suggested by prior research, specific character strengths are expected to be positively related to school satisfaction (e.g., love of learning, zest, gratitude, perseverance, and curiosity; see Weber and Ruch, 2012), enjoyment of learning (e.g., zest, love of learning, perseverance; see Weber et al., 2016), and academic self-efficacy (e.g., hope, love of learning, perseverance, and prudence; see Weber and Ruch, 2012).

(2) What are the relations between character strengths as endogenous input variables and school achievement as an outcome variable? It is expected that specific character strengths are positively related to school achievement (e.g., perseverance, self-regulation, and love of learning; see Weber and Ruch, 2012; Wagner and Ruch, 2015).

(3) What are the relations between school satisfaction, enjoyment of learning, and academic self-efficacy as process variables and school achievement as outcome variable? All three process variables are expected to be positively related to school achievement (for reviews see Huebner et al., 2014; Pekrun, 2014; Schunk and Dibenedetto, 2014).

(4) Finally, and of an exploratory nature: What is the interplay between character strengths as endogenous input variables, specific process variables (school satisfaction, enjoyment of learning, and academic self-efficacy), and school achievement as an outcome variable? Based on the aforementioned findings, it is expected that the relations between specific character strengths and school achievement are mediated by school satisfaction, enjoyment of learning, and academic self-efficacy. To gain a fine-grained overview of the interplay of the variables of interest, one character strength and one process variables were entered in each analysis.

## Materials and methods

### Participants

The sample consisted of *N* = 300 children and adolescents (61.30% girls; 38.70% boys). Their mean age was *M* = 13.20 years (*SD* = 2.03; ranging from 10 to 17 years). Most of them (93.30%) attended secondary schools in the highest academic track (e.g., those conferring eligibility for university education in Germany), 6.30% attended medium-track secondary schools (e.g., normal learning tempo, needed to enroll in a demanding apprenticeship in Germany), and 0.40% attended other types of schools. Most of the participants were German (94.30%); the remaining 5.70% reported nationalities such as Turkish (1.30%), Italian, Russian, and Croatian (each 0.70%), and other nationalities (2.30%).

### Measures

The *Revised, Brief VIA-Youth* (Park and Peterson, 2006; German adaptation by Weber and Harzer, 2013) was used for the self-assessment of the 24 character strengths included in the VIA classification (Peterson and Seligman, 2004). The Revised, Brief VIA-Youth consists of 96 items (i.e., 4 items per character strength). Five items are reverse-keyed. The measure uses a 5-point answer format (from 1 = not like me at all to 5 = very much like me). Sample items are “My friends ask for my opinion before they make important decisions” (perspective) and “I am viewed as someone who gets things done” (perseverance). This revised, brief version of the scale showed a satisfactory overlap with the longer VIA-Youth (e.g., VIA Institute on Character, 2019), which has proven to be a reliable and valid measure (e.g., Park and Peterson, 2006; Ruch et al., 2014).

The school-specific subscale of the *Multidimensional Students’ Life Satisfaction Scale* (MSLSS; Huebner, 1994) was used for the self-assessment of school satisfaction. This subscale consists of 8 items (3 of them reverse-keyed) utilizing a 6-point answer format (from 1 = strongly disagree to 6 = strongly agree). A sample item is “I like being in school.” The German translation of the MSLSS was used, which shows satisfactory reliability and factorial validity (Weber, 2016). Evidence for the reliability and validity of the original English version of the MSLSS has also been reported (e.g., Huebner, 1994; Weber and Huebner, 2015; Weber et al., 2015).

The *Enjoyment of Learning Scale* (Jerusalem et al., 2009), which is based on Pekrun’s academic emotions approach (Pekrun, 1993), was used for the self-assessment of students’ enjoyment of learning activities. This scale consists of 3 items, which use a 4-point answer format (from 1 = strongly disagree to 4 = strongly agree). A sample item is “I enjoy learning new things in class.” Evidence for the scale’s satisfactory reliability has been reported (e.g., Jerusalem et al., 2009).

The *Academic Self-Efficacy Scale* (Jerusalem and Satow, 1999) was used for the self-assessment of students’ beliefs about their ability to master tasks and challenges at school (i.e., academic self-efficacy). It consists of 7 items (one reverse-keyed) using a 4-point answer format (from 1 = strongly disagree to 4 = strongly agree). A sample item is “I can even master the difficult tasks at school if I try hard.” Evidence for satisfactory reliability and validity has been reported (e.g., Weber, and Ruch, 2012).

As a proxy for students’ *school achievement*, participants were asked to provide information from their latest school report card on two major subjects (i.e., language arts and mathematics) via the following two questions: “What was your last grade in German language arts? What was your last grade in mathematics?” Answers were given on a 6-point answer format (from 1 = fail to 6 = very good). A composite score was calculated by averaging these two grades. Research has shown that self-reported grades and actual grades from school records are highly related (e.g., Gray and Watson, 2002; Kuncel et al., 2005; Möller et al., 2006; Noftle and Robins, 2007; Sparfeldt et al., 2008), indicating that self-reported grades have satisfactory validity.

### Procedure

Prior to data collection, the study was approved by the relevant institutional review board. Participants were then recruited in German public schools. In accordance with human subjects guidelines (e.g., American Psychological Association, 2017), all children and adolescents participated voluntarily and provided both their personal assent as well as the informed consent of their parents or legal guardians. Web-based data collection^1^ was employed; participants were asked to complete the online survey at home to minimize time pressure bias while answering the survey. Participants were not paid for their services, but upon request, received written individualized feedback on their character strengths and written material explaining their individual results.

### Data analysis

**FIGURE 2:**
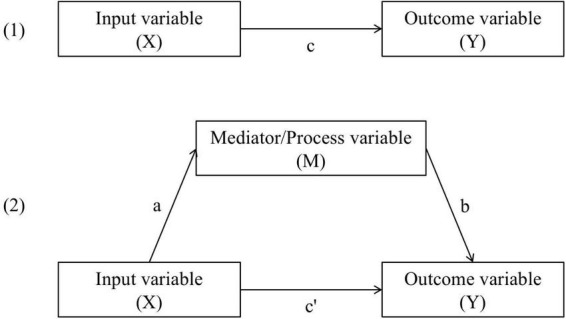
Total effect model **(1)** and basic mediation model **(2)** adapted from Hayes (2013).

All data analyses were computed using the statistical software package SPSS 24. All regression analyses and mediation analyses were based on the Model 4 template of PROCESS v2.16.3 for SPSS (Hayes, 2013). Furthermore, bias-corrected bootstrapped confidence intervals for the indirect effects were estimated in the mediation analyses utilizing this PROCESS template (*N* = 5,000 bootstrapped samples; 99% confidence level for confidence intervals). Figure 2 shows the calculated coefficients a, b, c, c′, and the indirect effect (a × b).

Because the VIA classification consists of 24 different character strengths, most of the research questions involved multiple tests of significance. A conservative level of significance of *p* < 0.001 was used when interpreting the findings from analyses involving the full list of 24 character strengths in order to correct for the number of significance tests.

## Results

### Preliminary results

Means, standard deviations, Cronbach’s alpha coefficients, zero-order correlations with participants’ age, and sex differences were computed for all key variables. Results for the 24 character strengths (input variables) are reported in Table 2. Table 3 presents the results for school satisfaction, enjoyment of learning, and academic self-efficacy (process variables) as well as for school achievement (outcome variable).

**TABLE 2:** Input variables: Means, standard deviations, and internal consistency (Cronbach’s alpha) of 24 character strengths, zero-order correlations of 24 character strengths with age (r_age_), and sex differences in 24 character strengths.

	Total sample (*N* = 300)				Males (*n* = 116)		Females (*n* = 184)			
Character strengths	*M*	*SD*	α	*r*_age_	*M*	*SD*	*M*	*SD*	*df*	*t*
Creativity	3.54	0.78	0.78	−0.34***	3.54	0.80	3.53	0.77	298	0.03
Curiosity	3.63	0.68	0.71	−0.19**	3.67	0.68	3.60	0.68	298	0.82
Judgment	3.47	0.71	0.78	−0.12*	3.57	0.70	3.40	0.71	298	2.01*
Love of learning	3.28	0.70	0.84	−0.19***	3.41	0.69	3.21	0.70	298	2.44*
Perspective	3.64	0.62	0.72	–0.07	3.61	0.63	3.65	0.62	298	–0.50
Bravery	3.60	0.66	0.73	−0.27***	3.56	0.67	3.63	0.66	298	–0.86
Perseverance	3.55	0.76	0.79	−0.34***	3.51	0.80	3.58	0.73	298	–0.79
Honesty	3.50	0.68	0.76	−0.19***	3.47	0.74	3.51	0.64	218	–0.49
Zest	3.77	0.73	0.82	−0.29***	3.82	0.69	3.74	0.75	298	0.87
Love	4.02	0.69	0.69	−0.12*	3.88	0.66	4.11	0.69	298	−2.93**
Kindness	4.02	0.60	0.74	−0.17**	3.87	0.66	4.12	0.54	208	−3.53***
Social intelligence	3.91	0.53	0.59	–0.05	3.94	0.57	3.90	0.51	298	0.60
Teamwork	4.01	0.51	0.60	–0.08	3.97	0.59	4.03	0.46	200	–0.93
Fairness	3.63	0.67	0.76	−0.27**	3.62	0.73	3.64	0.64	298	–0.26
Leadership	2.97	0.81	0.81	–0.01	3.00	0.80	2.96	0.82	298	0.42
Forgiveness	4.07	0.65	0.82	−0.19***	4.11	0.64	4.04	0.66	298	0.78
Modesty	3.67	0.68	0.54	–0.06	3.57	0.74	3.73	0.64	298	−1.98*
Prudence	3.44	0.71	0.76	–0.11	3.48	0.73	3.40	0.70	298	0.96
Self-regulation	3.49	0.68	0.68	–0.05	3.55	0.65	3.46	0.69	298	1.07
Appreciation	3.91	0.66	0.66	−0.17**	3.75	0.69	4.01	0.62	298	−3.41***
Gratitude	4.26	0.51	0.60	−0.22***	4.28	0.44	4.24	0.55	298	0.73
Hope	3.99	0.67	0.79	–0.06	4.11	0.61	3.92	0.69	298	2.38*
Humor	4.02	0.73	0.85	–0.07	4.03	0.76	4.01	0.71	298	0.22
Spirituality	2.88	1.02	0.82	−0.19***	2.88	1.03	2.88	1.01	298	–0.03

Age: Ranging from 10 to 17 years. *p < 0.05, **p < 0.01, ***p < 0.001.

**TABLE 3:** Process and outcome variables: Means, standard deviations, and internal consistency (Cronbach’s alpha) of school satisfaction, enjoyment of learning, academic self-efficacy, and school achievement, zero-order correlations of variables with age (r_age_), and sex differences in school satisfaction, enjoyment of learning, academic self-efficacy, and school achievement.

	Total sample (*N* = 300)				Males (*n* = 116)		Females (*n* = 184)			
Variables	*M*	*SD*	α	*r*_*age*_	*M*	*SD*	*M*	*SD*	*df*	*t*
***Processes***										
School satisfaction	4.22	0.88	0.90	−0.33***	4.33	0.89	4.15	0.87	298	1.70
Enjoyment of learning	2.85	0.61	0.73	−0.39***	2.89	0.61	2.82	0.61	298	1.09
Academic self-efficacy	3.01	0.43	0.72	−0.19***	3.10	0.40	2.96	0.44	298	2.88**
***Outcome***										
School achievement	4.62	0.77	—	−0.20***	4.54	0.76	4.67	0.77	298	–1.46

Age: Ranging from 10 to 17 years. **p < 0.01, ***p < 0.001.

Table 2 shows that the means of character strengths were numerically highest for gratitude (*M* = 4.26) and lowest for spirituality (*M* = 2.88), and that variability in the data is satisfactory (from *SD* = 0.51 for both teamwork and gratitude to *SD* = 1.02 for spirituality). These results are in line with earlier findings (e.g., Ruch et al., 2014). Most of the 24 character strength scales showed satisfactory reliability coefficients (i.e., 17 scales showed alpha coefficients > 0.70). Only modesty (α = 0.54), social intelligence (α = 0.59) as well as teamwork and gratitude (both αs = 0.60) exhibited coefficients below α = 0.65. All in all, the reliability coefficients of the 24 scales yielded a median of α = 0.76.

The average absolute correlation between the character strengths and participants’ age was *r* = |0.16| indicating that effect sizes were generally small in magnitude, but still evident. In line with earlier findings (e.g., Ruch et al., 2014), character strengths that were affected by participants’ age exhibited decreasing scores among older participants. Also sex differences in 24 character strengths were generally small in magnitude. Two noteworthy effects were found; that is, females were more likely to report higher scores in kindness and appreciation than males.

Table 3 shows the means and standard deviations of school satisfaction, enjoyment of learning, and academic self-efficacy, which were quite comparable with previously reported results (e.g., Jerusalem and Satow, 1999; Jerusalem et al., 2009; Weber, 2016). The findings for school achievement suggested that a broad range of grades was reported, indicating meaningful variability in the data. Reliability in terms of Cronbach’s alpha coefficients was satisfactory for all process variables (see Table 3). All process variables were affected by participants’ age, with decreasing scores among older students. Academic self-efficacy was affected by participants’ sex (i.e., males were more likely to report higher scores than females). Younger students were more likely to report higher levels of school achievement.

These preliminary analyses showed that several of the key variables appeared to be slightly affected by participants’ age and/or sex. Consequently, all subsequently computed analyses included age and sex as control variables.

### Main results

Multiple regression analyses were computed to answer research questions 1 and 2 on the relations between the 24 character strengths and school satisfaction, enjoyment of learning, academic self-efficacy as well as school achievement. The character strengths were entered as independent variables into the models (one character strength in each analysis). Additionally, age and sex were entered as independent variables into the analysis to control for their influence. The three process variables (i.e., school satisfaction, enjoyment of learning, academic self-efficacy) and the outcome variable (i.e., school achievement) were the dependent variables (one process variable in each analysis). Results are presented in columns a [for process variables] and c [for outcome variable] of Tables 4-6. The specific results are presented in the following subsections.

**TABLE 4:** Standardized coefficients of regression-based mediation analyses (Hayes, 2013) between character strengths (X, input variables) and school achievement (Y, outcome variable) mediated by school satisfaction (M, mediator/process variable).

Character strengths	a	b	c′	a × b [99% CI]	c
Creativity	0.31***	0.24***	0.03	0.07 [0.025; 0.129]	0.11
Curiosity	0.27***	0.25***	–0.02	0.07 [0.025; 0.132]	0.05
Judgment	0.25***	0.24***	0.05	0.06 [0.020; 0.122]	0.10
Love of learning	0.54***	0.17*	0.14*	0.09 [−0.018; 0.192]	0.23***
Perspective	0.43***	0.14*	0.22***	0.06 [−0.013; 0.147]	0.28***
Bravery	0.32***	0.21***	0.11	0.07 [0.014; 0.138]	0.18**
Perseverance	0.45***	0.14*	0.25***	0.06 [−0.005; 0.151]	0.31***
Honesty	0.30***	0.23***	0.07	0.07 [0.019; 0.132]	0.14*
Zest	0.47***	0.27***	–0.05	0.12 [0.045; 0.219]	0.08
Love	0.38***	0.21**	0.10	0.08 [0.016; 0.152]	0.18**
Kindness	0.39***	0.22***	0.07	0.09 [0.024; 0.171]	0.15**
Social intelligence	0.36***	0.25***	–0.01	0.09 [0.034; 0.177]	0.08
Teamwork	0.43***	0.21**	0.08	0.09 [0.016; 0.170]	0.17**
Fairness	0.38***	0.24***	0.03	0.09 [0.026; 0.169]	0.12*
Leadership	0.25***	0.21***	0.14*	0.05 [0.013; 0.117]	0.20***
Forgiveness	0.38***	0.19**	0.13*	0.07 [0.010; 0.159]	0.21***
Modesty	0.03	0.25***	0.08	0.01 [−0.029; 0.053]	0.09
Prudence	0.33***	0.21**	0.12	0.07 [0.018; 0.144]	0.18**
Self-regulation	0.32***	0.22***	0.08	0.07 [0.018; 0.148]	0.15**
Appreciation	0.37***	0.26***	–0.02	0.10 [0.040; 0.171]	0.07
Gratitude	0.37***	0.21**	0.10	0.08 [0.018; 0.154]	0.17**
Hope	0.46***	0.21**	0.07	0.10 [0.021; 0.194]	0.16**
Humor	0.10	0.26***	–0.10	0.03 [−0.011; 0.078]	–0.08
Spirituality	0.16**	0.23***	0.08	0.04 [0.006; 0.093]	0.12*

*N* = 300. a, b, c, and c′ in accordance with Figure 2. Analyses control for effects of age and sex. a × b = Completely standardized indirect effect (bias-corrected bootstrapped confidence intervals; *N* = 5000 bootstrapped samples). *p < 0.05, **p < 0.01, ***p < 0.001.

**TABLE 5:** Standardized coefficients of regression-based mediation analyses (Hayes, 2013) between character strengths (X, input variables) and school achievement (Y, outcome variable) mediated by enjoyment of learning (M, mediator/process variable).

Character strengths	a	b	c′	a × b [99% CI]	c
Creativity	0.36***	0.20**	0.03	0.07 [0.011; 0.140]	0.11
Curiosity	0.33***	0.22***	–0.02	0.07 [0.018; 0.145]	0.05
Judgment	0.20***	0.20**	0.07	0.04 [0.006; 0.100]	0.10
Love of learning	0.50***	0.12	0.17**	0.06 [−0.036; 0.159]	0.23***
Perspective	0.35***	0.11	0.24***	0.04 [−0.023; 0.109]	0.28***
Bravery	0.28***	0.17**	0.13*	0.05 [0.003; 0.113]	0.18**
Perseverance	0.32***	0.12*	0.27***	0.04 [−0.009; 0.104]	0.31***
Honesty	0.17**	0.19**	0.10	0.03 [0.004; 0.087]	0.14*
Zest	0.38***	0.21**	0.00	0.08 [0.012; 0.157]	0.08
Love	0.32***	0.17**	0.13*	0.05 [−0.001; 0.124]	0.18**
Kindness	0.35***	0.18**	0.09	0.06 [0.003; 0.137]	0.15**
Social intelligence	0.30***	0.21**	0.02	0.06 [0.012; 0.139]	0.08
Teamwork	0.29***	0.17**	0.12*	0.05 [0.003; 0.125]	0.17**
Fairness	0.28***	0.19**	0.07	0.05 [0.008; 0.119]	0.12*
Leadership	0.23***	0.17**	0.16**	0.04 [0.004; 0.105]	0.20***
Forgiveness	0.25***	0.17**	0.16**	0.04 [0.003; 0.105]	0.21***
Modesty	–0.08	0.22***	0.10	−0.02 [−0.069; 0.014]	0.09
Prudence	0.21***	0.18**	0.15*	0.04 [0.005; 0.100]	0.18**
Self-regulation	0.21***	0.19**	0.11	0.04 [0.005; 0.102]	0.15**
Appreciation	0.35***	0.22**	0.00	0.07 [0.018; 0.148]	0.07
Gratitude	0.28***	0.17**	0.13*	0.05 [0.006; 0.112]	0.17**
Hope	0.35***	0.17**	0.10	0.06 [0.002; 0.140]	0.16**
Humor	0.05	0.22***	–0.09	0.01 [−0.020; 0.057]	–0.08
Spirituality	0.19***	0.20**	0.08	0.04 [0.005; 0.095]	0.12*

*N* = 300. a, b, c, and c′ in accordance with Figure 2. Analyses control for effects of age and sex. a × b = Completely standardized indirect effect (bias-corrected bootstrapped confidence intervals; *N* = 5000 bootstrapped samples). *p < 0.05, **p < 0.01, ***p < 0.001.

**TABLE 6:** Standardized coefficients of regression-based mediation analyses (Hayes, 2013) between character strengths (X, input variables) and school achievement (Y, outcome variable) mediated by academic self-efficacy (M, mediator/process variable).

Character strengths	a	b	c′	a × b [99% CI]	c
Creativity	0.37***	0.50***	–0.08	0.18 [0.096; 0.274]	0.11
Curiosity	0.25***	0.49***	–0.07	0.12 [0.048; 0.214]	0.05
Judgment	0.20***	0.47***	0.01	0.09 [0.014; 0.180]	0.10
Love of learning	0.48***	0.47***	0.01	0.22 [0.138; 0.333]	0.23***
Perspective	0.52***	0.44***	0.05	0.23 [0.143; 0.340]	0.28***
Bravery	0.40***	0.48***	–0.01	0.19 [0.102; 0.287]	0.18**
Perseverance	0.46***	0.42***	0.11	0.19 [0.111; 0.281]	0.31***
Honesty	0.25***	0.47***	0.02	0.12 [0.046; 0.202]	0.14*
Zest	0.44***	0.54***	−0.15**	0.23 [0.142; 0.332]	0.08
Love	0.38***	0.47***	0.00	0.18 [0.102; 0.281]	0.18**
Kindness	0.32***	0.47***	0.00	0.15 [0.073; 0.249]	0.15**
Social intelligence	0.40***	0.52***	−0.13*	0.21 [0.123; 0.317]	0.08
Teamwork	0.36***	0.47***	0.00	0.17 [0.098; 0.270]	0.17**
Fairness	0.28***	0.47***	–0.01	0.13 [0.061; 0.215]	0.12*
Leadership	0.36***	0.46***	0.03	0.17 [0.088; 0.279]	0.20***
Forgiveness	0.32***	0.45***	0.06	0.14 [0.071; 0.230]	0.21***
Modesty	0.00	0.47***	0.09	0.00 [−0.071; 0.076]	0.09
Prudence	0.26***	0.45***	0.07	0.12 [0.044; 0.201]	0.18**
Self-regulation	0.30***	0.47***	0.01	0.14 [0.072; 0.232]	0.15**
Appreciation	0.32***	0.50***	–0.09	0.16 [0.075; 0.253]	0.07
Gratitude	0.38***	0.47***	–0.01	0.18 [0.100; 0.273]	0.17**
Hope	0.51***	0.52***	–0.10	0.27 [0.169; 0.386]	0.16**
Humor	0.14*	0.49***	−0.15**	0.07 [−0.007; 0.160]	–0.08
Spirituality	0.15*	0.46***	0.05	0.07 [−0.001; 0.147]	0.12*

*N* = 300. a, b, c, and c′ in accordance with Figure 2. Analyses control for effects of age and sex. a × b = Completely standardized indirect effect (bias-corrected bootstrapped confidence intervals; *N* = 5000 bootstrapped samples). *p < 0.05, **p < 0.01, ***p < 0.001.

#### Relations between character strengths and school satisfaction

Most character strengths (21 of 24) were highly significantly (*p* < 0.001) positively related to school satisfaction (except modesty, humor, and spirituality). The specific character strengths of love of learning, zest, hope, perseverance, perspective, and teamwork were the numerically highest correlates of school satisfaction (βs = 0.54 to 0.43; see column a of Table 4 for all coefficients).

#### Relations between character strengths and enjoyment of learning

Character strengths were significant correlates of enjoyment of learning, as 21 of 24 character strengths (except honesty, modesty, and humor) were highly significantly (*p* < 0.001) positively related to enjoyment of learning. The specific character strengths of love of learning, zest, creativity, perspective, kindness, appreciation, and hope were the numerically highest correlates of enjoyment of learning (βs = 0.50 to 0.35; see column a of Table 5 for all coefficients).

#### Relations between character strengths and academic self-efficacy

Character strengths emerged to be substantial correlates of academic self-efficacy, as 21 of 24 character strengths (except modesty, humor, and spirituality) were highly significantly (*p* < 0.001) positively related to academic self-efficacy. The specific character strengths of perspective, hope, love of learning, perseverance, zest, bravery, and social intelligence were the numerically highest correlates of academic self-efficacy (βs = 0.52 to 0.40; see column a of Table 6 for all coefficients).

#### Relations between character strengths and school achievement

The specific character strengths of perseverance, perspective, love of learning, forgiveness, and leadership, were highly significantly (*p* < 0.001) positive predictors of school achievement (βs = 0.31 to 0.20). The character strengths of bravery, love, teamwork, prudence, gratitude, and hope (all βs = 0.18 to 0.16) did not fulfill the conservative significance level of *p* < 0.001, but exhibited a tendency in the direction of a positive association with school achievement as well (see, for example, column c of Table 4 for all coefficients [identical coefficients are displayed in columns c of Tables 5, 6]).

#### Relations between school satisfaction, enjoyment of learning, academic self-efficacy, and school achievement

In order to answer research question 3 on the relations of school satisfaction, enjoyment of learning, and academic self-efficacy with school achievement, partial correlations (controlling for age and sex) were computed. All three process variables were substantially positively related to school achievement, with academic self-efficacy showing the strongest effect (*r* = 0.47, *p* < 0.001), followed by school satisfaction (*r* = 0.24, *p* < 0.001), and enjoyment of learning (*r* = 0.20, *p* < 0.001). That is, higher levels of these three process variables were associated with higher school achievement.

#### Relations between character strengths and school achievement mediated by school satisfaction, enjoyment of learning, or academic self-efficacy

Research question 4 addressed the assumption of the engine model of positive schooling (Harzer et al., 2021) that inputs (i.e., character strengths) have effects on outcomes (i.e., school achievement) mediated by processes (i.e., school satisfaction, enjoyment of learning, or academic self-efficacy). Therefore, a number of mediation analyses based on the Model 4 template of PROCESS v2.16.3 for SPSS (Hayes, 2013) were computed. Tables 4-6 (separate for the three specific process variables) present the effects of the input variables on the process variable (i.e., mediator variable) (column a), effects of the process variable on the outcome variable (column b), direct effects of the input variables on the outcome variable (column c′), indirect effects of the input variables on the outcome variable mediated by the process variable (column a × b), and total effects of the input variables on the outcome variable (column c).

##### School satisfaction as mediator/process variable

Table 4 shows that although the effect sizes were small in magnitude, 19 out of 24 analyses suggested a significant mediation effect of school satisfaction on the relation between character strengths and school achievement (see column a × b). No significant mediation effects were found for modesty or humor, which seemed reasonable as these two input variables were not related to the mediator variable of school satisfaction (see column a). Out of the character strengths that were highly significantly related to school achievement (i.e., love of learning, perspective, perseverance, leadership, and forgiveness), school satisfaction served as a mediator variable only for leadership and forgiveness. The links between school achievement and love of learning, perspective, and perseverance were not mediated by school satisfaction. The remaining character strengths did not exhibit a statistically significant direct link to school achievement (see column c′), but an indirect one mediated by school satisfaction. The indirect effects between zest, appreciation, and hope and school achievement were the numerically strongest of all 24 indirect effects (between 0.12 and 0.10). The indirect effects between kindness, social intelligence, teamwork, fairness, love, and gratitude and school achievement were between 0.09 and 0.08, while the indirect effects between creativity, curiosity, judgment, bravery, honesty, prudence, self-regulation, and spirituality and school achievement were 0.07 or lower, but still statistically significant.

##### Enjoyment of learning as mediator/process variable

Table 5 shows that although the effect sizes were small in magnitude, 18 out of 24 analyses suggested a mediation effect of enjoyment of learning on the relation between character strengths and school achievement (see column a × b). Again, no significant mediation effects were found for modesty or humor, as these two input variables did not relate to enjoyment of learning (see column a). Likewise, no significant mediation effect was found for love. Out of the character strengths that exhibited a significant direct relation to school achievement (i.e., love of learning, perspective, perseverance, leadership, and forgiveness), enjoyment of learning only served as a mediator variable for leadership and forgiveness. The links between love of learning, perspective, and perseverance and school achievement were not mediated by enjoyment of learning. The remaining character strengths did not exhibit a statistically significant direct link to school achievement (see column c′), but an indirect one mediated by enjoyment of learning. The indirect effects between zest, creativity, curiosity, and appreciation and school achievement were the numerically strongest of all 24 indirect effects (between 0.08 and 0.07). The indirect effects between kindness, social intelligence, and hope and school achievement were 0.06, whereas the indirect effects between judgment, bravery, honesty, teamwork, fairness, prudence, self-regulation, gratitude, and spirituality and school achievement were 0.05 or lower, but still statistically significant.

##### Academic self-efficacy as mediator/process variable

Table 6 shows that 21 out of 24 analyses suggested substantial mediator effects of academic self-efficacy on the relation between character strengths and school achievement (see column a × b). No significant mediation effects were found for modesty, humor, or spirituality, as these three input variables were not related to academic self-efficacy (see column a). Academic self-efficacy served as a mediator variable for all five character strengths that were directly related to school achievement (i.e., love of learning, perspective, perseverance, leadership, and forgiveness). Furthermore, the remaining character strengths did not exhibit a statistically significant direct link to school achievement (see column c′), but an indirect one mediated by academic self-efficacy. The numerically strongest indirect effects were found for the relations between hope, zest, perspective, love of learning, and social intelligence and school achievement (indirect effects between 0.27 and 0.21). The remaining significant indirect effects between character strengths and school achievement mediated by academic self-efficacy ranged between 0.19 and 0.09.

## Discussion

The present study was conducted to deliver further empirical evidence for an engine model describing various aspects relevant for positive schooling (Harzer et al., 2021). Therefore, relations were examined between character strengths as endogenous input variables; school satisfaction, enjoyment of learning, and academic self-efficacy as process variables; and school achievement as an outcome variable. Furthermore, the role of the process variables as mediators of the relations between the input variables and outcome variable was explored. Hence, the present study extends earlier research findings on character strengths’ role in the schooling context (e.g., Park and Peterson, 2006; Weber and Ruch, 2012; Shoshani and Slone, 2013; Wagner and Ruch, 2015; Weber et al., 2016). This section discusses the study’s main results with an eye to the proposed research questions.

### Main findings

The *first research question* focused on the relations between character strengths (as input variables) and school satisfaction, enjoyment of learning, and academic self-efficacy (as process variables). As expected, character strengths were positively associated with all three process variables. This is in line with Peterson and Seligman’s (2004) assumption that a good character – which manifests in character strengths – is a relevant human resource for various positive aspects of life like satisfaction, happiness, fulfilment, and success.

More specifically, the most substantial associations were exhibited between character strengths and students’ academic self-efficacy beliefs, with higher levels of most character strengths associated with higher levels of academic self-efficacy beliefs. This means that character strengths as positive personality traits are supportive resources that help students believe in their own capabilities when confronted with challenging tasks in class. This is in line with earlier findings suggesting that character strength are strong predictors of individuals’ self-efficacy beliefs (Weber and Ruch, 2012; Ruch et al., 2014). Specifically, the character strengths of perspective (i.e., having ways of looking at the world that make sense to oneself and to other people; Peterson and Seligman, 2004) and hope (i.e., expecting the best in the future and working to achieve it; Peterson and Seligman, 2004) showed the numerically strongest positive associations with academic self-efficacy beliefs.

Students’ character strengths were also meaningfully associated with indicators of wellbeing at school, specifically school satisfaction (i.e., students’ cognitive evaluation of the perceived quality of school experiences; e.g., Huebner, 1994) and enjoyment of learning (i.e., a positive emotion associated with achievement activities; e.g., Pekrun, 1993). Higher levels of character strengths are generally associated with higher school satisfaction and higher enjoyment of learning. Indeed, the character strength love of learning seems particularly relevant for these two variables, which makes sense because students who report higher levels of love of learning want to learn new things – everywhere and all the time (in school but also at home, with friends, etc.; Peterson and Seligman, 2004). Consequently, possessing higher levels of this schooling-relevant personality trait seems crucial for experiencing these two process variables reflecting wellbeing on a cognitive (school satisfaction) and an emotional level (enjoyment of learning).

The *second research question* focused on the relations between character strengths and school achievement. School achievement is a very central outcome variable in the context of schooling and has often been studied in relation to students’ cognitive abilities (e.g., Mackintosh, 1998; Deary et al., 2007). However, it has been shown that personality traits are also important predictors of school achievement; for example, students’ conscientiousness and persistence of motive seem to be relevant personality traits for success at school (e.g., De Raad and Schouwenburg, 1996; Noftle and Robins, 2007). Persistence is included in the set of 24 character strengths examined in this study (i.e., perseverance; Peterson and Seligman, 2004). The enormous advantage of the present study is that it applied a multidimensional model consisting of 24 positive personality traits (Peterson and Seligman, 2004) instead of focusing on only a single trait. This offers the opportunity to study the relations between individuals’ personality and school achievement in a very comprehensive and detailed way.

As expected and in line with earlier findings suggesting positive relations between character strengths and school achievement (e.g., Park and Peterson, 2006; Weber and Ruch, 2012; Wagner and Ruch, 2015), in the present study five character strengths (i.e., perseverance, perspective, love of learning, forgiveness, and leadership) exhibited noteworthy and meaningful positive associations with students’ achievement at school. All five of these character strengths appear to be supportive of students’ dealing with tasks and challenges at school, as shown by the following brief definitions:

*Perseverance*: Working diligently and hard to finish what one has started; “getting it out the door”; taking pleasure in completing tasks; determination to pursue future goals and not giving up easily (Peterson and Seligman, 2004).

*Perspective*: Having a meaningful view of life; viewed by others as being wise; others value one’s opinion very much; listening to others well and then giving them sensible and good advice (Peterson and Seligman, 2004).

*Love of learning*: Having a desire to learn a lot about life and the world; liking to learn new things, everywhere and all the time (i.e., in school but also at home, with friends, etc.); mastering new skills, topics, and bodies of knowledge (Peterson and Seligman, 2004).

*Forgiveness*: Forgiving those who have done wrong; accepting the shortcomings of others; giving people a second chance; not being vengeful; forgiving others who have hurt one (Peterson and Seligman, 2004).

*Leadership*: Encouraging a group of which one is a member to get things done; organizing group activities and seeing that they happen; managing to “keep the peace” in a group (Peterson and Seligman, 2004).

As shown in these definitions, perseverant students and students who love to learn are particularly able to succeed in their learning tasks. Students with more perspective are better able to listen and integrate information conveyed by the teacher. Finally, students possessing higher levels of forgiveness and leadership function better in groups. Therefore, it makes sense that these five character strengths are directly associated with higher achievement at school.

The *third research question* focused on the relations among school satisfaction, enjoyment of learning, and academic self-efficacy (as process variables) and school achievement (as outcome variable). The results of the present study are in line with earlier findings (e.g., Huebner et al., 2014; Pekrun, 2014; Schunk and Dibenedetto, 2014). As expected, academic self-efficacy was positively associated with school achievement, that is, students’ who believe in their capability to master school-related tasks and challenges are more likely to succeed in school. This positive relation also implies – as the direction of a correlation is not fully clear in cross-sectional data – that students with high levels of success experience more self-efficacy in the academic context. In addition, wellbeing-related processes like school satisfaction and enjoyment of learning were related to school achievement. The correlational data suggest that either students who are satisfied with school and enjoy learning are more likely to succeed in school or vice versa. Although these findings exhibited significant but only small effect sizes in the present study, they are relevant for a better understanding of positive schooling broadly construed, meaning schooling that does not exclusively focus on mastery-related processes like academic self-efficacy, but also considers wellbeing-related processes.

The *fourth research question* addressed the schooling-related engine model’s assumption that inputs are related to outcomes mediated by relevant processes (Harzer et al., 2021). Overall, the mastery-related process of academic self-efficacy emerged as a relatively clear mediator between character strengths and school achievement. Furthermore, the wellbeing-related processes of school satisfaction as well as enjoyment of learning mediated most of the relations between character strengths and school achievement, but the effect sizes were smaller than for academic self-efficacy.

In line with Pajares and Schunk (2001), the present study underscores the process character of academic self-efficacy, as this variable substantially mediated the relations between most of the character strengths (inputs) and school achievement (outcome). It appears that students who possess higher levels of character strengths (e.g., hope, zest, perspective, love of learning, social intelligence) are more likely to believe in their own capabilities, which is in turn associated with greater success at school.

Research on the mediating role of school satisfaction is still relatively sparse (e.g., Huebner et al., 2014), but the present study adds further knowledge to this topic. School satisfaction mediated the relations between most of the character strengths and school achievement. For example, higher levels of the character strength teamwork help students work well together with other students (e.g., while solving a task in class) or find friends and become a member of an in-group at school. Such a positive attitude toward working/being together with others in the school context is very likely associated with students’ satisfaction with their school experiences, which in turn should be supportive of better school performance. Conversely, it appears plausible that students who are not part of an in-group at school are very likely to have a hard time there, which should in turn be associated with lower levels of school satisfaction and thus lower school achievement.

Additionally, emotion-related characteristics like positive emotions/affect have been proposed to have a mediating function (e.g., Lyubomirsky et al., 2005; Pekrun, 2014), as described in the schooling-related engine model (Harzer et al., 2021). The findings of the present study suggest that enjoyment of learning is a mediator of the relations between specific character strengths (inputs) and school achievement (outcome). For example, students who possess higher levels of the character strength prudence exhibit a self-management style that helps them effectively achieve their long-term goals because they think and care about their future, form long-term goals, are able to resist self-defeating impulses, and engage in a flexible and reflective style of thinking (Peterson and Seligman, 2004). It makes sense that higher levels of prudence are relevant for the experience of positive feelings toward learning at class (i.e., enjoyment of learning), because such students know that learning is important for achieving their long-term goals. Such an attitude is very likely to result in better achievement at school. Although we know that there is a positive relation between prudence and positive feelings at school (Weber et al., 2016), and although the present study found a small (but significant) mediation effect for the relation between prudence and school achievement (mediated by enjoyment of learning), further studies are needed to understand these relations more deeply.

Another relevant finding concerned the processes of school satisfaction and enjoyment of learning: Significant indirect relations were found between specific character strengths and school achievement even though no direct relations were found. However, it is not necessary to show such a direct link before testing for a possible mediation effect (e.g., Hayes, 2013). For example, a noteworthy indirect effect emerged between zest and school achievement, with school satisfaction as the mediator. This is interesting because zest was not significantly directly related to school achievement. Nevertheless, zest plays a significant role for school satisfaction (Weber and Ruch, 2012; Weber, 2018) and for satisfaction in general (e.g., Park et al., 2004; Ruch et al., 2014; Weber, 2021), and school satisfaction plays a significant role for school achievement, as has been shown in the present study. As another example, curiosity is not directly related to school achievement, but is related to enjoyment of learning, which is in turn positively related to school achievement. It would be surprising if such a relevant aspect of human personality (i.e., being curious and interested) was not related to achievement at school, but the relationship is clearer now: Students who are more curious enjoy learning activities at school, which is in turn highly relevant for performing well in school. Hence, realizing and studying such indirect paths appears quite useful for a nuanced understanding of the interrelations between relevant key variables in the schooling context, as examined in the present study. The engine model of positive schooling (Harzer et al., 2021) makes such interrelations more apparent.

### Limitations and future research

The findings of the present study are promising. However, some limitations need to be discussed and further research is needed. *First*, the German language version of the Revised, Brief VIA-Youth scale was employed for the first time in the present study; hence, more studies are needed to provide further data on both its reliability and validity. However, as certain results are fully in line with prior research utilizing the longer version of the VIA-Youth (e.g., Weber and Ruch, 2012; Ruch et al., 2014), the presented results appear trustworthy. In addition, four scales of the Revised, Brief VIA-Youth questionnaire yielded unsatisfactory internal consistencies (below 0.65); however, this did not impact the main results of the present study. Nevertheless, further research is needed to determine whether this is an effect of the present sample or a limitation of this measure. *Second*, the present study utilized self-ratings of students’ school achievement. Although the data showed satisfactory variability, future studies should also consider collecting grades from students’ official school records to eliminate possible bias in the data. However, the presented results can be evaluated as trustworthy, as research shows high accuracy of self-reported grades (e.g., Gray and Watson, 2002; Kuncel et al., 2005; Noftle and Robins, 2007). For example, in German samples (a) correlations between self-reported and actual grades are around *r* = 0.90, (b) mean differences are small, and (c) correlations of self-reported grades with other variables do not differ substantially from the correlations of actual grades with other variables (e.g., Möller et al., 2006; Sparfeldt et al., 2008). *Third*, to establish further generalizability, more studies are needed examining these research questions cross-nationally and cross-culturally. Furthermore, more heterogeneous samples are needed. In addition, the study of specific “sub-samples” with respect to aspects like age group, education level (e.g., low vs. high learning tempo), school type (e.g., public vs. private schools), socioeconomic status, and intellectual capabilities can also provide information on generalizability. *Fourth*, the present research was conducted to explore the mediation effects of three separate process variables. However, such process variables are substantially interrelated, hence, future research is needed that examines more complex models adding two or more process variables simultaneously to the analyses. This would help to further study the reciprocity between different process variables (e.g., Magnano et al., 2020) in our model (Harzer et al., 2021). Furthermore, Lent and Brown (2008) postulated a model considering the interplay between variables like personality, self-efficacy, and satisfaction (among others). Combinations of our model (Harzer et al., 2021) and other models (e.g., Lent and Brown, 2008) will help to better understand the complexity of positive schooling. *Finally*, the present study utilized a cross-sectional design examining the interplay among character strengths, school satisfaction, enjoyment of learning, academic self-efficacy, and achievement at school. This means that causality cannot be established from the present data. To examine the relations between inputs, processes, and outcomes in a deeper and more nuanced way, (short-term and long-term) longitudinal studies with two (or more) measurement points need to be designed. That would also help to further validate the engine model of positive schooling (Harzer et al., 2021).

### Conclusion and implications

From a *theoretical* perspective, the present study’s findings contribute to a better understanding of the role of positive personality traits (i.e., character strengths) in positive processes like school satisfaction, enjoyment of learning, and a self-efficacious handling of challenges at school. Unraveling the associations between various types of variables, the present study focused on both (1) direct relations between the different components of an engine model of positive schooling (e.g., relations between input and process variables or relations between process and outcome variables), and (2) results that consider the complexity of this schooling-related model (i.e., examining inputs, processes, and outcomes simultaneously) (Harzer et al., 2021). Furthermore, although the present results do not allow for causal interpretations, all reported results clearly underline the positive interplay between the key variables studied here. The present study untangled the phenomenon that character strengths (although not always directly related to school achievement) are significant for school achievement because they are related to relevant mediator variables like school satisfaction, enjoyment of learning, or academic self-efficacy, which are in turn related to school achievement. These findings underscore the important role of character strengths in the schooling context.

From a *practical* perspective, the findings meaningfully demonstrate that students’ personalities in terms of character strengths as endogenous resources clearly matter at school, and that certain character strengths emerged as more relevant than others for experiencing school satisfaction, enjoyment of learning, or academic self-efficacy. As students differ in their character strengths profiles, it is important to realize that individual differences in character strengths might be directly related to individual differences in the processes students experience, which might in turn have an impact on students’ performance at school. Consequently, character strengths programs need to focus on fostering individual strengths instead of forcing the same “prescribed” required character strengths upon students (Lavy, 2020). Moreover, the engine model of positive schooling (Harzer et al., 2021) highlights that school achievement as an outcome should not be evaluated without taking further relevant factors into account (i.e., inputs like teachers’ behaviors and students’ personality; processes like satisfaction, enjoyment, and self-efficacy). Such a comprehensive model offers the opportunity to identify various aspects affecting good but also poor performance at school. For example, one starting point for teachers to ignite a positive cycle within the engine model of positive schooling on student-level might be their self-compassion and autonomy-supportive motivating styles (Moè and Katz, 2020) as exogenous input variables for students’ processes like academic self-efficacy beliefs and outcomes like school success. This knowledge might be relevant for various practitioners in the schooling context (e.g., principals, teachers, school psychologists), but also for parents and for the design of intervention programs to foster and develop positive schooling broadly construed.

## Data availability statement

The datasets presented in this article are not readily available because participants of the study did not agreed for their data to be shared publicly, so data unfortunately cannot be shared publicly. Requests should be directed to MW, m.weber@mail.de.

## Ethics statement

The studies involving human participants were reviewed and approved by the Institutional Review Board, University of South Carolina, Columbia, SC, United States. Informed consent to participate in this study was provided by the participants’ legal guardian/next of kin.

## Author contributions

MW contributed to the conception and design of the study. Both authors organized the database, performed the statistical analyses, and contributed to the final version of the manuscript.

## Footnotes

1
http://www.unipark.com/en/
